# Obesity Paradox and Surgical Evacuation for Chronic Subdural Hematoma

**DOI:** 10.7759/cureus.24002

**Published:** 2022-04-10

**Authors:** David R Hallan, Zachary Freedman, Elias Rizk

**Affiliations:** 1 Neurosurgery, Penn State Health Milton S. Hershey Medical Center, Hershey, USA

**Keywords:** mortality rate, outcomes, burrhole drainage, obesity paradox, obesity, subdural hematoma, neurosurgery

## Abstract

Background: Chronic subdural hematoma (cSDH) has a number of risk factors for recurrence, and some studies suggest obesity is one of them. Yet obesity has been shown to have a positive survival benefit in many diseases such as ischemic stroke, chronic obstructive pulmonary disease, percutaneous coronary intervention, and mechanical thrombectomy. Therefore, we sought to determine if obesity provided a mortality benefit in patients with cSDH undergoing burr hole drainage or craniotomy.

Methods: We performed a retrospective database analysis using a multi-institutional (TriNetX) database looking at obese versus non-obese patients with cSDH undergoing surgical drainage. Our primary endpoint was mortality. Secondary endpoints included seizure, pulmonary embolism (PE), myocardial infarction (MI), cerebral infarction, deep vein thrombosis (DVT), tracheostomy, and percutaneous endoscopic gastrostomy (PEG). These were looked at to obtain a better idea of prognosis. Cohorts were propensity score-matched for confounders, using the greedy-nearest neighbor algorithm with a caliper of 0.1 pooled standard deviations. Kaplan-Meier survival curves were also developed, and hazard ratios were calculated. Chi-square analysis was performed on categorical variables.

Results: A total of 1,849 patients were identified as obese with a drainage procedure, while 12,371 were identified as non-obese. Some 1,746 patients remained in each group after propensity score matching. Thirty-day survival rates were 88.08% in the obese vs. 83.82% in the non-obese cohorts, 90-day survival 85.15% vs. 79.35%, 365-day survival at 80.89% vs. 71.90%, and five-year survival at 64.75% vs. 54.84% (p < 0.0001). The risk difference was -8.02% (95% confidence interval, Cl -11.02, -5.021%); relative risk, RR 0.757, 95% Cl (0.67, 0.841); odds ratio, OR 0.676 (0.583, 0.783); p < 0.0001). Seizures, ventilator dependence, MI, cerebral infarction, tracheostomy, and PEG rates were all non-significant. Obese patients had a higher rate of PE (7.90% vs. 4.47%, p < 0.0001) and DVTs (12.37% vs. 10.02%, p = 0.0278).

Conclusions: Obesity in patients with cSDH undergoing surgical evacuation is associated with decreased mortality but higher rates of DVT and PE.

## Introduction

Obesity, a condition in which an excessive amount of fat negatively affects a person’s health, has been a relentless pandemic for the past several decades with estimates totaling 30% of the world’s population who reach the age of 60 being considered obese [1]. This pandemic comes with adverse outcomes in patients, especially in the central nervous system. Evidence has shown that obesity negatively affects the central nervous system through free fatty acid-induced lipotoxicity, oxidative stress, endoplasmic reticulum stress, sympathetic alterations, and metabolic dysfunction [2].

Chronic subdural hematoma (cSDH), an unfortunately common central nervous system condition in the elderly, has several risk factors for recurrence, including obesity [3]. Despite this, obesity has been shown to have a positive mortality benefit in many diseases such as ischemic stroke, chronic obstructive pulmonary disease, percutaneous coronary intervention, and mechanical thrombectomy [4-5]. This paradox, known as the obesity paradox, was first introduced in 2002 by Gruberg et al., who described the increased mortality benefit in obese individuals in coronary artery disease [6]. Since then, numerous conditions have been documented to have an increased mortality benefit in the obese population. To our knowledge, this has not been previously studied in cSDH.

While most research has been done to identify the obesity paradox in cardiopulmonary and cardiovascular diseases, the evidence of the obesity paradox in neurosurgical procedures, especially skull-based procedures, remains poor. This may be due to the location of adipose tissue, with less in and around the skull and brain. As a result, obesity is a minor consideration for neurosurgical cranial procedures.

The objective of this study was to determine if obesity provided a mortality benefit in patients with cSDH undergoing burr hole drainage or craniotomy.

## Materials and methods

We used a de-identified database network (TriNetX) to retrospectively query via ICD-10 and current procedural terminology codes to evaluate all patients with a diagnosis of atraumatic non-acute subdural hematoma with subsequent burr hole drainage or craniotomy and obesity (cohort 1) vs. no obesity (cohort 2). Data came from 56 health care organizations (HCOs) spanning six countries. Data include demographics, diagnoses, medications, laboratory values, genomics, and procedures. The identity of the HCOs and patients is not disclosed to comply with ethical guidelines against patient re-identification. Because of the database's federated nature, an IRB waiver has been granted. Our use of this database and its validity was informed by previous literature, and exact details of the network have been previously described [7-10]. The index date was set at the date of drainage.

The medical information included age at index date, as well as sex, race, and comorbidities of hypertension, acute kidney injury, diabetes, ischemic heart disease, heart failure, atrial fibrillation, disorders of lipoprotein metabolism and other lipidemias, obesity, history of nicotine dependence, chronic respiratory disease, cirrhosis, alcohol abuse or dependence, and peripheral vascular disease, were recorded up to the date of the index date. Codes can be found in Table 1. Antiplatelet and anticoagulation medications were likewise recorded. There were too few patients with disseminated intravascular coagulation, hereditary factor VIII and IX deficiency, and Von Willebrand's disease to include. Our primary outcome of interest was mortality, with secondary outcomes of mechanical ventilation, tracheostomy, PEG tube placement, seizure, pulmonary embolism (PE), myocardial infarction (MI), ischemic stroke (IS), and deep venous thrombosis (DVT). These outcomes were looked at over a period of five years, with interval analysis at 30, 90, 180, and 365 days. Patients were propensity score-matched into cohorts using the greedy-nearest neighbor algorithm with a caliper of 0.1 pooled standard deviations, in order to correct for the above factors and possible confounders. Analysis was performed on unmatched and matched cohorts. Hazard ratios were calculated using R's survival package v3.2-3 and validated, comparing the output to SAS version 9.4. Chi-square analysis was performed on categorical variables. The significance level was set at a p-value less than 0.05. 

## Results

A total of 1,849 patients were identified as obese with a drainage procedure, while 12,371 were identified as non-obese. Some 1,746 patients remained in each group after propensity score matching. After matching, age at index was 61.0+-16.6 and 62.1+-20.6 for Cohorts 1 and 2, respectively. Some 62.486% of Cohort 1 were male, and 61.798% in Cohort 2. Some 73.368% vs. 72.623% of patients were White, 15.750% vs. 16.495% were Black or African American, and 0.7450% vs. 1.031% were Asian. Baseline demographics and characteristics are shown in Table 1.

**Table 1:** Baseline demographics and characteristics after propensity score matching.

		Before matching			After matching		
Code	Diagnosis	Cohort 1, n (%)	Cohort 2, n (%)	Std diff.	Cohort 1, n (%)	Cohort 2, n (%)	Std diff.
AI	Age at Index	61.13 (100)	59.00 (100)	-	60.95 (100)	62.09 (100)	-
2106-3	White	1350 (73.37)	8240 (70.54)	0.063	1282 (73.43)	1285 (73.59)	0.0039
M	Male	1136 (61.74)	7877 (67.43)	0.12	1090 (62.43)	1047 (59.97)	0.051
F	Female	704 (38.26)	3801 (32.54)	0.12	656 (37.57)	699 (40.03)	0.051
2054-5	Black or African American	294 (15.98)	1623 (13.89)	0.059	274 (15.69)	266 (15.24)	0.013
2131-1	Unknown race	166 (9.02)	1542 (13.20)	0.13	162 (9.28)	154 (8.82)	0.016
2028-9	Asian	13 (0.71)	223 (1.91)	0.11	13 (0.75)	26 (1.49)	0.071
I10-I16	Hypertensive diseases	1580 (85.87)	6382 (54.64)	0.73	1486 (85.11)	1495 (85.62)	0.015
E78	Disorders of lipoprotein metabolism and other lipidemias	1134 (61.63)	3336 (28.56)	0.70	1040 (59.57)	1047 (59.97)	0.0082
E08-E13	Diabetes mellitus	844 (45.87)	2099 (17.97)	0.63	753 (43.13)	749 (42.89)	0.0046
R53	Malaise and fatigue	794 (43.15)	2481 (21.24)	0.48	712 (40.78)	760 (43.53)	0.056
R40	Somnolence, stupor, and coma	730 (39.67)	3152 (26.98)	0.27	669 (38.32)	692 (39.63)	0.027
I20-I25	Ischemic heart diseases	703 (38.2`)	2375 (20.33)	0.40	641 (36.71)	633 (36.25)	0.0095
N17-N19	Acute kidney failure and chronic kidney disease	666 (36.19)	1863 (15.95)	0.47	593 (33.96)	583 (33.39)	0.012
J40-J47	Chronic lower respiratory diseases	613 (33.32)	1773 (15.18)	0.43	544 (31.16)	563 (32.25)	0.023
R13	Aphagia and dysphagia	503 (27.34)	1964 (16.81)	0.26	470 (26.92)	471 (26.98)	0.0013
I48	Atrial fibrillation and flutter	524 (28.48)	1865 (15.97)	0.30	466 (26.69)	444 (25.43)	0.029
I50	Heart failure	484 (26.30)	1315 (11.26)	0.39	424 (24.28)	434 (24.86)	0.013
Z87.891	Personal history of nicotine dependence	476 (25.87)	1395 (11.94)	0.36	420 (24.06)	439 (25.14)	0.025
F17	Nicotine dependence	403 (21.90)	1606 (13.75)	0.21	366 (20.96)	368 (21.08)	0.0028
R63	Symptoms and signs concerning food and fluid intake	268 (14.57)	1031 (8.83)	0.18	247 (14.15)	248 (14.20)	0.0016
F10.1	Alcohol abuse	187 (10.16)	1098 (9.40)	0.025	178 (10.19)	184 (10.54)	0.011
I73	Other peripheral vascular diseases	209 (11.36)	499 (4.27)	0.27	174 (9.97)	174 (9.97)	0.00
F10.2	Alcohol dependence	152 (8.26)	844 (7.23)	0.039	141 (8.08)	148 (8.48)	0.015
K74	Fibrosis and cirrhosis of liver	83 (4.51)	279 (2.39)	0.112	71 (4.066)	80 (4.58)	0.025
1191	Aspirin	872 (47.39)	2804 (24.01)	0.50	789 (45.19)	798 (45.70)	0.010
11289	Warfarin	421 (22.88)	898 (7.69)	0.43	362 (20.73)	364 (20.85)	0.0028
8410	Alteplase	142 (7.72)	393 (3.36)	0.19	123 (7.05)	112 (6.42)	0.025
1364430	Apixaban	73 (3.97)	168 (1.44)	0.16	64 (3.67)	65 (3.72)	0.003
1114195	Rivaroxaban	67 (3.64)	141 (1.21)	0.159	59 (3.38)	50 (2.86)	0.029
31500	Intubation, endotracheal, and emergency procedure	295 (16.03)	1554 (13.30)	0.077	273 (15.64)	278 (15.92)	0.0079

Thirty-day survival rates were 88.08% in the obese vs. 83.82% in the non-obese cohorts, 90-day survival 85.15% vs. 79.35%, 365-day survival at 80.89% vs. 71.90%, and five-year survival at 64.75% vs. 54.84%. The risk difference was -8.02% (95% Cl -11.02, -5.021%); RR 0.757, 95% Cl (0.67, 0.841); OR 0.676 (0.583, 0.783); p < 0.0001.

**Figure 1:**
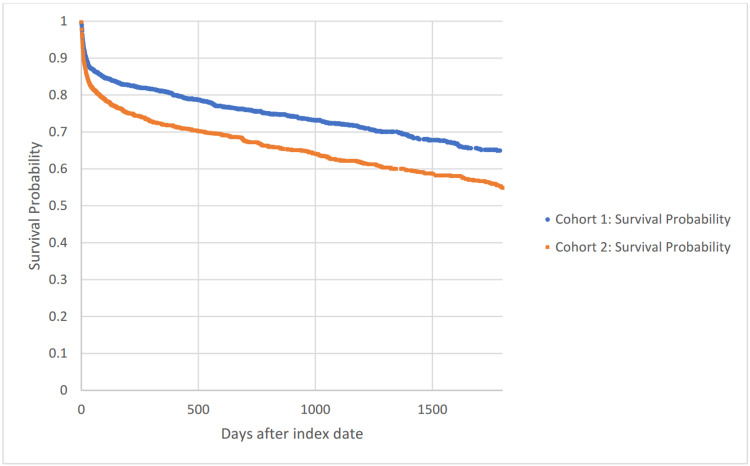
Kaplan-Meier survival analysis for outcome: deceased.

Figure 1 shows a Kaplan-Meier survival curve for outcome deceased through five years comparing Cohorts 1 and 2. The hazard ratio was 0.756, with 95% CI (0.667, 0.857), p < 0.0001.

Table 2 shows outcomes after propensity score matching.

**Table 2:** Outcomes after propensity score matching. DVT, deep venous thrombosis; PEG, percutaneous endoscopic gastrostomy; PE, pulmonary embolism; MI, myocardial infarction; CI, confidence interval; OR, odds ratio

Outcome	Cohort 1, n (%)	Cohort 2, n (%)	OR (95% CI)	p-value
Mortality	437 (25.03)	544 (31.16)	0.74 (0.64,0.86)	<0.0001
Ventilator dependence	197 (11.28)	190 (10.88)	1.04 (0.83,1.29)	0.7059
Tracheostomy	147 (8.42)	116 (6.64)	1.29 (1.00,1.66)	0.0468
PEG	167 (9.57)	165 (9.45)	1.01 (0.81,1.27)	0.9081
Seizures	700 (40.09)	731 (41.87)	0.93 (0.81,1.06)	0.2861
PE	138 (7.90)	73 (4.18)	1.97 (1.47,2.63)	<0.0001
MI	122 (6.99)	124 (7.10)	0.98 (0.76,1.27)	0.8948
Ischemic stroke	184 (10.54)	171 (9.79)	1.09 (0.87, 1.35)	0.4666
DVT	216 (12.37)	176 (10.08)	1.26 (1.02,1.56)	0.0320

Seizures, dependence on a respirator, MI, cerebral infarction, tracheostomy, and PEG were non-significant. Obese patients had a higher rate of PE (7.90% vs. 4.47%, p=<0.0001) and DVTs (12.37% vs. 10.02%, p=0.0278). Some 36% of those with PE were also in the mortality group for Cohort 1.

## Discussion

Our results demonstrate a significant decrease in mortality in patients who are obese that have undergone a burr hole procedure, as seen in the Kaplan-Meier survival curve in Figure 1. Furthermore, the absolute risk difference between Cohort 1 and Cohort 2 after the procedure continued to increase until one year with a -4.26% at 30 days, -5.8% at 90 days, and -7.19% at 180 days, where it eventually plateaued between -9% and -8% between years one and five.

Additionally, comparing these cohorts for secondary outcomes showed a significant increase in a tracheostomy procedure (29.2%), PE (96.7%), and DVT (25.9%) following a burr hole procedure in the obese cohort. Secondary outcomes that were not significant between groups were ventilator dependence, PEG surgery, seizures, MI, and ischemic stroke.

These findings of an increased survival rate of burr hole or craniotomy procedures following cSDH in obese individuals continue to shed light on the obesity paradox that was first described almost 20 years ago.

Literature review shows that the obesity paradox may exist for other neurological conditions such as intracerebral hemorrhage and subarachnoid hemorrhage [11-12]. However, the role of the obesity paradox in craniotomy or craniectomy procedures continues to be controversial. Studies that look at obese patients undergoing these procedures have focused primarily on functional outcomes rather than mortality. Research has shown an increase in 30-day readmission risk, and an increase in surgical site infections among other functional outcomes [13-14]. The studies that do look at mortality outcomes have shown no significant difference in mortality with obese patients undergoing craniotomy or craniectomy for a variety of procedures [15-16]. To our knowledge, this is the first study looking at cSDH, craniotomy, obesity, and mortality.

In addition to the mortality benefit, we also found a significant increase in tracheostomy procedures, PE, and DVT. This may be due to the previously known associations of tracheostomy and emboli with obesity [17-18]. On the other hand, mortality benefits may be explained by obesity in a few ways. Weight-loss related to illness may cause obesity to be protective. A higher body mass index (BMI) could include a higher reserve of muscle and adipose to draw upon in this situation. Furthermore, if obesity is more likely to be associated with cSDH incidence, then perhaps obese patients have better outcomes because they are less likely to have other, unknown, risk factors associated with the development of cSDH that we were not able to match for. Furthermore, we do not know the ratio of muscle to adipose tissue in our obese patients, and it could be that these patients who are doing well have a high muscle mass.

Overall, this data shows the existence of the obesity paradox in obese patients with csDH undergoing surgical intervention. They also were shown to have higher rates of DVT, PE, and tracheostomy than their non-obese counterparts. This study could be expanded on using prospective longitudinal cohort analysis to assess the validity of the data presented.

Our analysis was not without limitations. The major limitation of this study was that it was retrospective. Furthermore, due to the nature of the database, we were unable to collect patient-level data on specific outcomes. We were unable to report on radiology information. We do not have information on the type of diagnostic test used for confirmation of disease. We do not know how large the subdural hematoma was. The data collected were for billing purposes, not for clinical use, and thus much clinical information is missing. In addition, some misidentification is inevitable in database studies.

## Conclusions

The role of the obesity paradox in craniotomy and other neurosurgical procedures is controversial. In this study, we demonstrate that in patients with csDH undergoing surgical evacuation, obesity is associated with decreased mortality but higher rates of DVT and PE. This is despite the fact that obesity increases the risk of cSDH formation. To our knowledge, this is the first study looking at cSDH, craniotomy, obesity, and mortality, and continues to shed light on the obesity paradox that was first described almost 20 years ago.
